# Randomized phase III trial of consolidation therapy with bortezomib–lenalidomide–Dexamethasone (VRd) vs bortezomib–dexamethasone (Vd) for patients with multiple myeloma who have completed a dexamethasone based induction regimen

**DOI:** 10.1038/bcj.2016.55

**Published:** 2016-07-29

**Authors:** S J Jacobus, S V Rajkumar, M Weiss, A K Stewart, E A Stadtmauer, N S Callander, L M Dreosti, M Q Lacy, R Fonseca

**Affiliations:** 1 Dana Farber Cancer Institute – ECOG-ACRIN Biostatistics Center, Boston, MA, USA; 2 Mayo Clinic, Rochester, MN, USA; 3 ThedaCare, Appleton, WI, USA; 4 Mayo Clinic in Arizona, Scottsdale, AZ, South Africa; 5 University of Pennsylvania, Philadelphia, PA, USA; 6 University of Wisconsin, Madison, WI, USA; 7 University of Pretoria, Pretoria, South Africa

Long-awaited results from the the Southwest Oncology Group (SWOG) trial comparing bortezomib–lenalidomide-dexamethasone (VRd) versus lenalidomide-dexamethasone (Rd) as induction treatment for previously untreated multiple myeloma (MM) patients were recently presented (S0777).^1^ Results justified the early adoption of VRd in the frontline setting over 5 years ago by the NCCN based on Level 2A evidence according to their guidelines.^2^ The randomized study E1A05 also sought to evaluate VRd superiority over a doublet (Vd), but in the consolidation setting. The trial was closed to enrollment prematurely due to slow accrual. Results reported here include 48 enrolled patients.

Patients with symptomatic MM who had completed a minimum of one cycle and maximum of six cycles of dexamethasone-based induction therapy were eligible provided no more than 8 weeks had elapsed since induction end. Patients were stratified by prior lenalidomide-dexamethasone use during induction and by complete response (CR) status at registration. Patients in the VRd arm received bortezomib (V) 1.3 mg/m^2^ IV d1, 4, 8 and 11, lenalidomide (R) 15 mg/day PO days 1–14 plus dexamethasone (d) 40 mg/day PO d1, 8 and 15 every 21 days for 8 cycles; patients in the Vd arm received bortezomib 1.3 mg/m^2^ IV d1, 4, 8 and 11 plus dexamethasone 40 mg/day PO d1, 8, 15 every 21 days for 8 cycles. The study was designed to detect a 50% improvement in median progression-free survival (PFS) from 21 months (m) to 31.5 m using a stratified log-rank test given 392 patients, 212 failures, 82% power and 2.5% one-sided Type I error.

Descriptive statistics were used to characterize patients at study entry. Response evaluation was based on the International Myeloma Working Group (IMWG) response criteria.^3^ Toxicity was evaluated according to CTCAEv3, including only events with at least possible treatment attribution. Response and toxicity rates were estimated with 95% exact binomial confidence intervals. The odds ratio for objective response (OR), defined as partial response (PR) or better, was estimated with 95% CIs. PFS was defined as the time from randomization to the earliest documentation of disease progression (PD) or death from any cause. Patients who were alive without evidence of PD were censored at the date of last disease assessment. Time-to-event distributions were estimated using the Kaplan-Meier (KM) method and compared using the log-rank test in all randomized patients according to assigned treatment (intention to treat, ITT).^4^ Cox proportional hazards regression was used to assess treatment hazard ratio (HR, VRd/Vd).^5^ Restricted mean survival time, which represents the area under the survival curves when cutting follow-up at a certain time, was also calculated.^6^ Quality of life (QoL) was evaluated using the FACT-Neurotoxicity Trial Outcome Index, which is the sum of the FACT physical, functional and neurotoxicity instruments (25 questions, score 0–100). A minimal absolute difference in mean change score between arms of 6-8 or half observed standard deviation (s.d.) was considered clinically significant.^7^ QoL was measured at baseline, during treatment (cycle 5), end of treatment (cycle 8 or earlier) and in long-term follow-up (months 9, 12, 15 and 18 post registration).

Between January 2008 and May 2010, 48 patients from 15 institutions (85% ECOG) were randomized, with equal allocation to VRd (*n*=23) and Vd (*n*=25). Of the study cohort, 56% were ⩾65 years, 44% female, 85% white, 58% PS>0 and 19% ISS Stage III. Median prior dose of dexamethasone was 640 mg on both arms, with 22 patients receiving Rd. On study, mean duration of treatment was 6.5 cycles. 56% patients completed eight cycles (VRd 65%, Vd 48%). The main reason for early discontinuation was due to adverse event/complications (66%). Three patients underwent transplant within 6 m of ending treatment, but no further data on subsequent therapy were collected. The primary response analysis data set comprised 32 patients (16/arm) with confirmed measurable disease at baseline. Response rates were higher on the VRd arm, including OR rates of 81% vs 63% (odds ratio 2.6 (95% CI (confidence interval): 0.52, 13.0)) and ⩾very good partial response rates of 63% vs 19%. Sensitivity analysis excluding only patients not in CR at registration (*n*=3) added 14 more unevaluable patients to the existing 3. In this subset, OR and ⩾very good partial response rates on VRd and Vd, respectively, were 59% vs 43% and 45% vs 13%. Grade 3 or higher treatment-related, non-hematological toxicity rates between arms were similar (VRd 65 vs Vd 64%) (Table 1). Grade 3-4 neurologic/pain/gastrointestinal toxicity rates were 35%/13%/9% on VRd vs 28%/16%/16% on Vd. Five second primary malignancies were reported (3 Vd, 2 VRd). Median PFS was similar: VRd 20 m (95% CI: 11–43 m) vs Vd 18 m (95% CI: 9–37 m) (Figure 1). The PFS HR was 0.96 (95% CI: 0.53–1.75), with 44 failures (92%) overall. Median survival follow-up was 72 months (52% of patients have died). Median OS was 69 m on VRd and 60 m on Vd. The OS HR was 0.77 (95% CI: 0.35–1.70). Five-year OS probability was 65% (95% CI: 48–88%) on VRd and 52% (95% CI: 36–76%) on Vd. Setting follow-up at 60 m, patients on VRd survived for 50.1 m on average, a survival savings of 4.8 m (95% CI: −4.4 to −14 m). The FACT Ntx TOI mean change from baseline to 6 m post treatment end (primary) was −7.9 VRd (*n*=12) vs −2.6 Vd (*n*=8) with a s.d. of change of 15 in both arms. The mean change to treatment end was −14.9 VRd (*n*=19) vs −8.0 Vd (*n*=20). Patients on average experienced −3 to −5 and −1 to −2 point decreases over treatment in FACT physical and functional score, respectively, on the VRd arm compared with little change on the Vd arm. The two arms had parallel adverse trends over treatment in the FACT Ntx domain: −4 to −8 VRd vs −3 to −6 Vd.

The trial results in terms of comparing treatments are limited because of the small sample size. Our study demonstrated that VRd was not substantially less tolerated than Vd, but appeared inferior in terms of QoL driven by differences in FACT physical and less so functional domains comprising the FACT Ntx TOI. There was a trend towards improved long-term survival on the VRd arm with 5-year OS probability 65% (95% CI: 48–88%) vs Vd 52% (95% CI: 36–76%), echoing S0777 results. PFS was overlapping between arms in our study and short relative to S0777 due to the consolidation setting and lack of maintenance treatment on protocol. VRd neurologic/pain toxicity as well as OR rates paralleled S0777 results despite lower lenalidomide dosing. Response analyses show that accurate representation of efficacy can be compromised due to the stringent nature of coding disease at baseline and in follow-up, wherein a missing test can lead to ineligible/unevaluable status. Not only are response rates often underestimated but also randomization is not upheld.

The premature closure of E1A05 due to weak accrual was not uncharacteristic of clinical trial research in the cooperative group setting. Research has shown that a phase III therapeutic trial takes on average 2.5 years from formal concept review to submission and that length of development time is adversely associated with accrual performance.^8, 9^ Activated in September 2007, E1A05 was on par, with about a 2-year development time. The final E1A05 study design centered on comparing a two- vs three-drug regimen using the most promising novel agents of the time.^10^ At concept initiation, however, the goal was to compare VRd against a regimen that included VRd and high-dose therapy with autologous stem cell transplant (HDT), but a design with transplant randomization was not palatable for reviewers at that time. The value of chemotherapy-based consolidation for myeloma compared with HDT in terms of OS is still unclear, but is being tested in an international trial. The E1A05 experiment became obsolete fairly quickly, with VRd superiority a foregone conclusion based initially on reports of a Phase I/II study and confirmed later in other small studies showing unprecedented results for VRd.^11, 12, 13^

At ASCO 2013 the NCI MM Accrual Working Group published 10 Barriers to Accrual (BtA) and presented strategies to overcome these.^14^ E1A05 represents an example of BtA 2 (spectrum of available treatment options), 6 (competing trials including S0777 among others) and 7 (lack of interest in a trial evaluating commercially available agents only). Adding in the larger context of weak patient accrual metrics in the US, the problem was intensified.^15^ If E1A05 had met its accrual rate and event targets, definitive information on VRd would have been available as early as March 2011. The NCI has made serious efforts to improve the process of clinical trial activation with the establishment of more aggressive timelines to be met (OEWG) as well as the creation of a central IRB (CIRB). Furthermore, the development and deployment of disease-specific steering committees within the NCI was pursued with the intent to coordinate clinical trial development and, specifically, to prevent competing clinical trials within the NCI Clinical Trial Network. The cooperative group oncology clinical research enterprise provides the platform for independent, practice-changing research in MM, but the operational environment is very challenging and initiatives to enhance accrual need to continue.

## Figures and Tables

**Figure 1:**
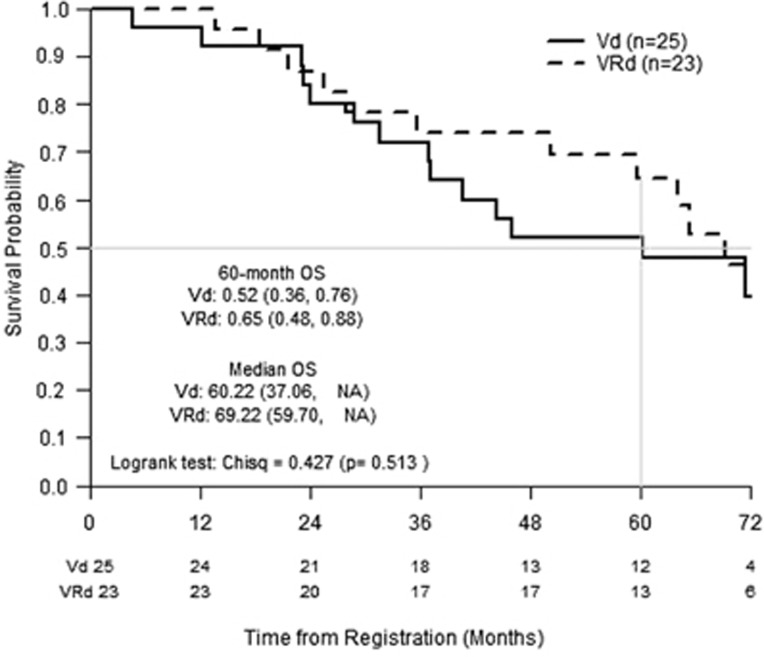
Overall Survival by Treatment Arm.

**Table 1:** Incidence of Treatment-Related Toxicity by Treatment Arm

*Toxicity Category*	*VRd (*n=*23)*			*Vd (*n=*25)*		
	*Grade*			*Grade*		
N *(%)*	*1,2*	*3*	*4*	*1,2*	*3*	*4*
Blood	3 (13%)	-	4 (17%)	7 (28%)	1 (4%)	3 (12%)
Constitutional	5 (22%)	3 (4%)	-	5 (20%)	5 (20%)	-
Gastrointestinal	3 (13%)	2 (9%)	-	4 (16%)	4 (16%)	-
Infection	-	1 (4%)	-	-	4 (16%)	-
Metabolic	2 (9%)	2 (9%)	-	2 (8%)	-	-
Neurologic	4 (17%)	8 (35%)	-	3 (12%)	6 (24%)	1 (4%)
Pain	2 (9%)	3 (13%)	-	2 (8%)	3 (12%)	1 (4%)
WORST DEGREE (Non-Hematologic)	2 (9%)	15 (65%)	-	4 (16%)	15 (60%)	1 (4%)

## References

[bib1] Durie BG, Hoering A, Rajkumar SV, Abidi MH, Epstein J, Kahanic SP et al. Bortezomib, Lenalidomide and Dexamethasone vs. Lenalidomide and Dexamethasone in patients (Pts) with previously untreated multiple myeloma without an intent for immediate autologous stem cell transplant (ASCT). Results of the randomized phase III trial SWOG S0777. Blood (ASH Annual Meeting 177 Abstracts) 2015, 653. 10.1038/s41408-020-0311-8 PMC7214419 32393732

[bib2] National Comprehensive Cancer network (NCCN) Guidelines Version 4. Multiple Myeloma. 2015.

[bib3] Durie BG, Harousseay JL, Miquel JS, Blade J, Barlogie B, Anderson K et al. International uniform response criteria for multiple myeloma. Leukemia 2006; 20: 1467–1473. 16855634 10.1038/sj.leu.2404284

[bib4] Kaplan EL, Meier P. Nonparametric estimation from incomplete observation. J Am Stat Assoc 1958; 53: 457–481.

[bib5] Cox DR. Regression models and life tables. J Royal Stat Soc B 1972; 34: 181–220.

[bib6] Uno H, Claggett B, Tian L, Inoue E, Gallo P, Miyata T et al. Moving beyond the hazard ratio in quantifying the between-group difference in survival analysis. JCO 2014; 32: 2380–2385. 10.1200/JCO.2014.55.2208 PMC4105489 24982461

[bib7] Norman GR, Sloan JA, Wyrwich KW. Interpretation of changes in health-related quality of life: the remarkable universality of half a standard deviation. Med Care 2003; 41: 582–592. 12719681 10.1097/01.MLR.0000062554.74615.4C

[bib8] Dilts D, Cheng S, Crites J, Sandler A, Doroshow J. Phase III clinical trial development: a process of chutes and ladders. CCR 2010; 16: 5381–5389. 10.1158/1078-0432.CCR-10-1273 PMC3058405 21062928

[bib9] Cheng S, Dietrich M, Dilts D. A sense of urgency: evaluating the link between clinical trial development time and the accrual performance of cancer therapy evaluation program (NCI-CTEP) sponsored studies. CCR 2010; 16: 5557–5563. 10.1158/1078-0432.CCR-10-0133 PMC3050630 21062929

[bib10] Smith A, Wistloff F, Samson D. Guidelines on the diagnosis and management of multiple myeloma 2005. Br J Haematol 2005; 132: 410–451. 10.1111/j.1365-2141.2005.05867.x 16412016

[bib11] Richardson P, Jagannath S, Raje N, Jakubowiak A, Lonial S, Avigan D et al. Lenalidomide, Bortezomib, and Dexamethasone (Rev/Vel/Dex) as front-line therapy for patients with multiple myeloma (MM). Preliminary results of a Phase 1/2 study. Blood 2007; 110: 187.

[bib12] Roussel M, Avet-Loiseau H, Moreau P. Frontline therapy with bortezomib, lenalidomide, and dexamethasone (VRD) induction followed by autologous stem cell transplantation, VRD consolidation and lenalidomide maintenance in newly diagnosed multiple myeloma patients: primary results of the IFM 2008 phase II study. Blood 2010; 116: 624.

[bib13] Kumar S, Flinn I, Richardson PG. Randomized, Multicenter, phase 2 study (EVOLUTION) of combinations of bortezomib, dexamethasone, cyclophosphamide, and lenalidomide in previously untreated multiple myeloma. Blood 2012; 119: 4375–4382. 22422823 10.1182/blood-2011-11-395749

[bib14] Weiss M, Gertz MA, Little RF, Rajkumar V, Jacobus SJ, Abonour R et al. Strategies to overcome barriers to accrual (BtA) to NCI-sponsored clinical trials: a project of the NCI-Myeloma Steering Committee Accrual Working Group (NCI-MYSC AWG). ASCO Annu Meet Proc 2013; 31, Abstr 8592.

[bib15] Institute of MedicineA National Cancer Clinical Trials System for the 21st Century: Reinvigorating the NCI Cooperative Group Program. The National Academies Press: Washington, DC, USA, 2010. 25032387

